# O.2.1-7 Young people’s perspectives on integrating targeted physical activity in substance use treatment: implications for practice

**DOI:** 10.1093/eurpub/ckad133.112

**Published:** 2023-09-11

**Authors:** Lisa Klamert, Melinda Craike, Gillinder Bedi, Susan Kidd, Alexandra Parker

**Affiliations:** Institute for Health & Sport, Victoria University, Australia; Centre for Youth Mental Health, The University of Melbourne, Australia; Institute for Health & Sport, Victoria University, Australia; Mitchell Institute for Education and Health Policy, Victoria University, Australia; Orygen; Centre for Youth Mental Health, The University of Melbourne, Australia; Tweed Byron Mental Health Acute Care Service; lee.klamert@vu.edu.au; Institute for Health & Sport, Victoria University, Australia; Orygen; Centre for Youth Mental Health, The University of Melbourne, Australia

## Abstract

**Purpose:**

Problematic substance use (SU) in young people is a global health concern and leads to difficulties in key life areas. Rates of problematic SU are highest in young people aged 15-25 years. Physical activity (PA) may reduce problematic SU (ie., alcohol, tobacco, illicit use), and provide a less stigmatizing treatment option. Yet, research on this treatment approach in this population remains limited, targeted PA is rarely routinely implemented in substance treatment for young people, and global policy lacks responsivity to this health priority.

**Method:**

A quantitative research survey (n = 145 young Australians) and a qualitative focus group (n = 4) were conducted and analyzed. Eligibility criteria included: i) aged 15-25 years; ii) moderate or severe risk of substance-related health and other problems (assessed by WHO ASSIST); and iii) prior access of SU treatment or willing to engage in future treatment. The purpose of this research was to explore young people’s perspectives on the (1) acceptability, (2) experienced barriers, and (3) preferences regarding the integration of PA as an adjunct intervention in existing treatment services.

**Results:**

Survey data showed that 97.8% of young people found preference-driven PA interventions acceptable within SU treatment practice. A further 94.9% believed that PA-based interventions are appropriate and effective (95.6%) for reducing their SU, and 94.9% expressed their willingness to engage in these interventions. Barriers to PA were exercise-induced fatigue, cost of PA, lack of motivation, existing substance use, and lack of PA guidance. Interview data showed a clear preference for highly tailored, planned but unsupervised interventions, with frequent check-ins. Additional preferences were clear directives, highly passionate and motivating facilitators and a focus on behavior change. Preference-driven PA interventions were perceived to provide a sense of personal accomplishment and perceived care from service providers.

**Conclusion:**

PA is perceived to be acceptable within the treatment of SU among young people and may offer a non-stigmatizing adjunct to existing treatments. Factors that might influence translation of PA interventions into practice and policy were identified in the preferences of young people involved in this study, highlighting the importance of including young people as key stakeholders in intervention design and delivery.

